# Glucomannan-mediated facile synthesis of gold nanoparticles for catalytic reduction of 4-nitrophenol

**DOI:** 10.1186/1556-276X-9-404

**Published:** 2014-08-20

**Authors:** Zhao Gao, Rongxin Su, Renliang Huang, Wei Qi, Zhimin He

**Affiliations:** 1 State Key Laboratory of Chemical Engineering, School of Chemical Engineering and Technology, Tianjin University, Tianjin 300072, People’s Republic of China; 2 School of Environmental Science and Engineering, Tianjin University, Tianjin 300072, People’s Republic of China; 3 Collaborative Innovation Center of Chemical Science and Engineering (Tianjin), Tianjin 300072, People’s Republic of China

**Keywords:** Gold nanoparticles, Catalysis, Composites, Glucomannan, 4-Nitrophenol

## Abstract

A facile one-pot approach for synthesis of gold nanoparticles with narrow size distribution and good stability was presented by reducing chloroauric acid with a polysaccharide, konjac glucomannan (KGM) in alkaline solution, which is green and economically viable. Here, KGM served both as reducing agent and stabilizer. The effects of KGM on the formation and stabilization of as-synthesized gold nanoparticles were studied systematically by a combination of UV-visible (UV-vis) absorption spectroscopy, transmission electron microscopy, X-ray diffraction, dynamic light scattering, and Fourier transform infrared spectroscopy. Furthermore, the gold nanoparticles exhibited a notable catalytic activity toward the reduction of 4-nitrophenol to 4-aminophenol.

## Background

In the recent years, noble metal nanoparticles, especially gold nanoparticles (AuNPs), have attracted great interest and wide attention. AuNPs have proven to be a versatile platform in many areas such as catalysis, biosensing, optoelectronics, biological imaging, and therapeutic techniques [1-3]. Recently, the preparation and potential applications of AuNPs are becoming increasingly popular among researchers due to their distinctive optical properties, particularly tuneable surface plasmon resonance. Up to now, a number of chemical and physical methods for synthesis of metal nanoparticles have been reported, such as chemical reduction, electro-reduction, photo-reduction, and heat evaporation [4-6]. In most cases, the synthetic processes either involve the use of borohydride, hydrazine, citrate, etc. or require rather complex procedures or rigorous conditions, followed by surface modification with some protecting ligands like thiols and oleic acid. Thus, both toxicity and high cost make these materials less promising in industrial and biological applications.

To address these problems, biosynthesis of biological materials has received considerable attention. Compared to traditional methods, biosynthesis has many advantages by decreasing the use of toxic chemicals in the process and eliminating risks in industrial, pharmaceutical, and biomedical applications. To date, a broad range of biological materials has been introduced for the biosynthesis of metal nanoparticles including phytochemicals (polyphenol extract, catechin, lemongrass leaf extract, aloe extract, and fruit extracts) [7-13], microorganisms (bacteria and yeast) [14-16], protein [17,18], peptide [19,20], and polysaccharide [21-24].

Among the various biological materials, polysaccharides are emerging as an important natural resource for the synthesis of metal nanoparticles. In such processes, polysaccharides usually act as a reducing agent or stabilizer because of their special structure and properties. Since Raveendran et al. proposed a completely green method for preparation of silver nanoparticles with starch [23], many researchers have investigated the effects and mechanism of various polysaccharides on the formation of metal nanoparticles, such as cellulose, chitosan, alginic acid, hyaluronic acid, and agarose [21-25]. Konjac glucomannan (KGM), a kind of natural polysaccharide, has been widely used for its several valuable functions in healthcare and pharmacology, such as obesity suppression, tumor suppression, and treatment of cough, hernia, and skin disorders [26,27]. The studies on the applications of konjac glucomannan have been extended greatly from food and food additives to various fields [28,29]. Herein, we explore the use of KGM in the preparation of nanosized materials and thus further promote its application in nanotechnology.

**Figure 1:**
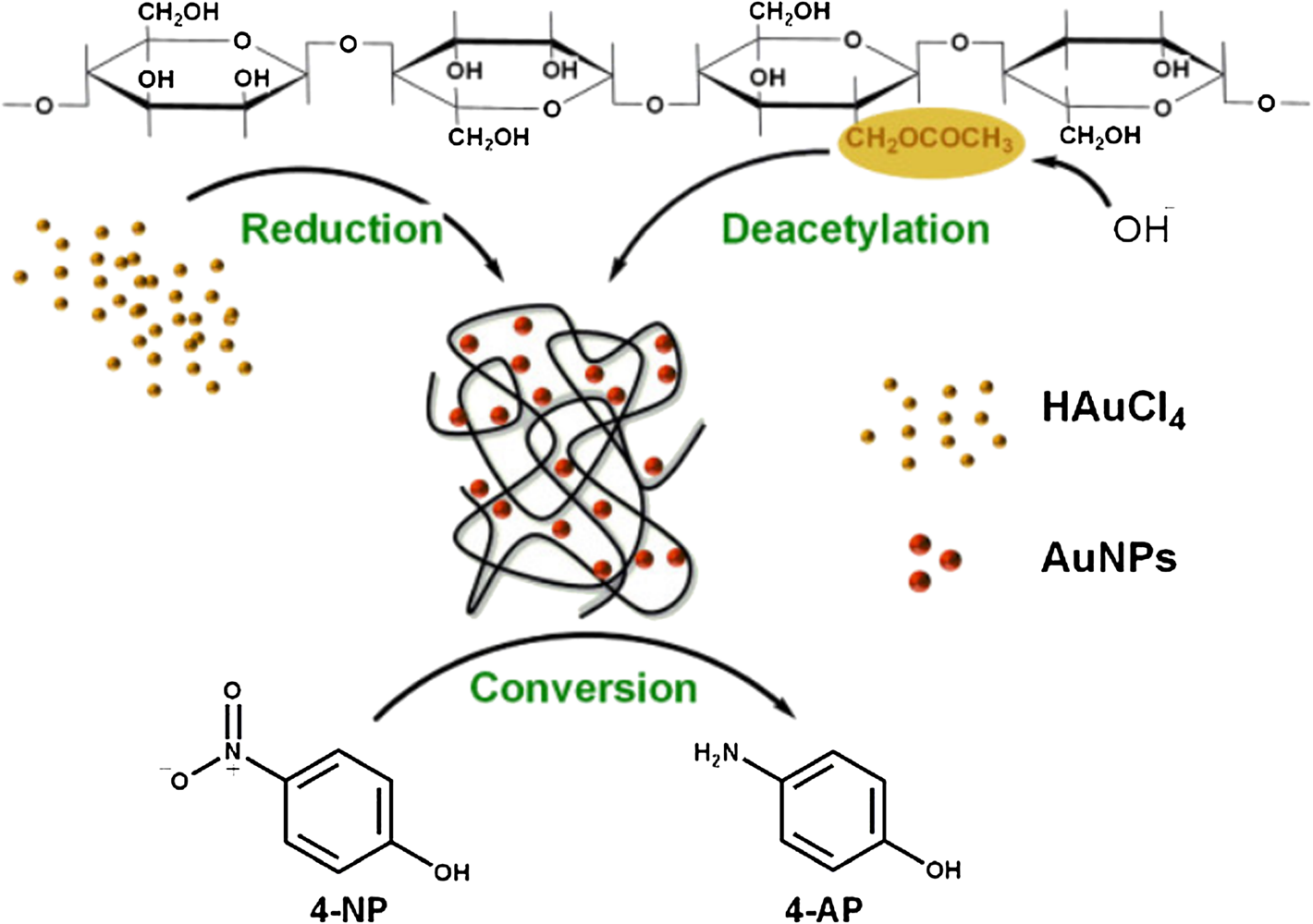
Schematic plot illustrating the formation and stabilization of AuNPs using konjac glucomannan.

In the present study, konjac glucomannan was introduced for the facile synthesis of gold nanoparticles, both as reducing agent and stabilizer (Figure 1). The synthesized gold nanoparticles were characterized in detail by transmission electron microscopy (TEM), X-ray diffraction (XRD), dynamic light scattering (DLS), and Fourier transform infrared (FTIR) spectroscopy. Furthermore, the catalytic activity of the gold nanoparticles was investigated by the reduction of *p*-nitrophenol (4-NP) to *p*-aminophenol (4-AP). It should be noted that Konjac glucomannan, as an abundant natural polysaccharide, could be easily gained from Konjac plant tubers at low cost. Meanwhile, the gold nanoparticles reduced in the aqueous KGM solution exhibit great stability and dispersibility due to specific properties of KGM.

## Methods

### Materials

Chloroauric acid (HAuCl_4_ · 4H_2_O, 99.9%) was purchased from Aladdin (Shanghai, China). Purified konjac glucomannan was obtained from Shengtemeng Konjac Powder Co. (Sichuan, China). All solutions were prepared in double-distilled water, and all glassware used was rinsed with aqua regia solution (HCl/HNO_3_, 3:1) and then washed with double-distilled water before use. All other common reagents and solvents used in this study were of analytical grade.

### Synthesis of AuNPs in aqueous solution with KGM

KGM powders (0.25 g) were dispersed in double-distilled water (100 mL) by stirring for 1 h at room temperature, and then the solution was held at 80°C for 1 h. The preparation of gold nanoparticles is quite straightforward. In a typical preparation, sodium hydroxide solution (0.4 mL, 1 M) was added to KGM solution (20 mL, 0.25 wt%) under stirring, and then aqueous HAuCl_4_ (2 mL, 10 mM) solution was introduced. The mixture was incubated at 50°C for 3 h. The obtained gold nanoparticles were collected by centrifugation and washed thoroughly with DI water.

### Characterization

All UV-visible (UV-vis) spectra were recorded on a Pgeneral TU-1810 spectrophotometer (Purkinje Inc., Beijing, China) with 1-cm quartz cells. At different time intervals, aliquots of the solution were taken out and the samples were cooled to ambient temperature and then tested immediately. The morphology of the prepared gold nanoparticles in KGM solutions was examined with a JEOL JEM-2100 F transmission electron microscope (TEM, JEOL Inc., Tokyo, Japan) operated at an acceleration voltage of 200 kV. After ultrasonication for approximately 10 min in a bath sonicator, samples were prepared by placing a drop of aqueous gold nanoparticles onto a 300-mesh carbon-coated copper grid, and the grids were left to air dry at ambient temperature. Dynamic light scattering measurements were performed using a Brookhaven ZetaPlus Nanoparticle Size Analyzer instrument (Brookhaven Instruments Corporation, Holtsville, NY, USA) equipped with a 633-nm laser. The intensity of light scattered was monitored at a 90° angle. The XRD data was collected on a D/MAX 2500 diffractometer (Cu Kα radiation, *λ* = 1.5406 Å; Rigaku Co., Tokyo, Japan) at 100 mA and 40 kV. The sample was scanned over a 2*θ* range of 10° to 90° with a step size of 0.02° 2*θ* and a scan rate of 1 step/s. Fourier transform infrared (FTIR) spectra were recorded on a Nicolet-560 FTIR spectrometer (Nicolet Co., Madison, WI, USA) with 20 scans and a resolution of 2 cm^-1^ in the range of 400 to 4,000 cm^-1^. Freeze drying under vacuum was applied overnight to get the very dry gold nanoparticles, and then the samples were deposited on the surface of a KBr plate.

### Catalytic activity of gold nanoparticles

The catalytic activity of AuNPs was studied using sodium borohydride reduction of 4-NP as a model system. The reaction was completed in a quartz cell with a 1-cm path length. In a typical catalysis reaction, 15 μL of 10 mM 4-NP solution was mixed with 3 mL of 10 mM NaBH_4_ solution while stirring. Immediately after 15 μL of the prepared AuNP solution was added to the mixture, the reaction was monitored by a UV-vis spectrophotometer.

## Results and discussion

### Synthesis of AuNPs in aqueous KGM solution

**Figure 2:**
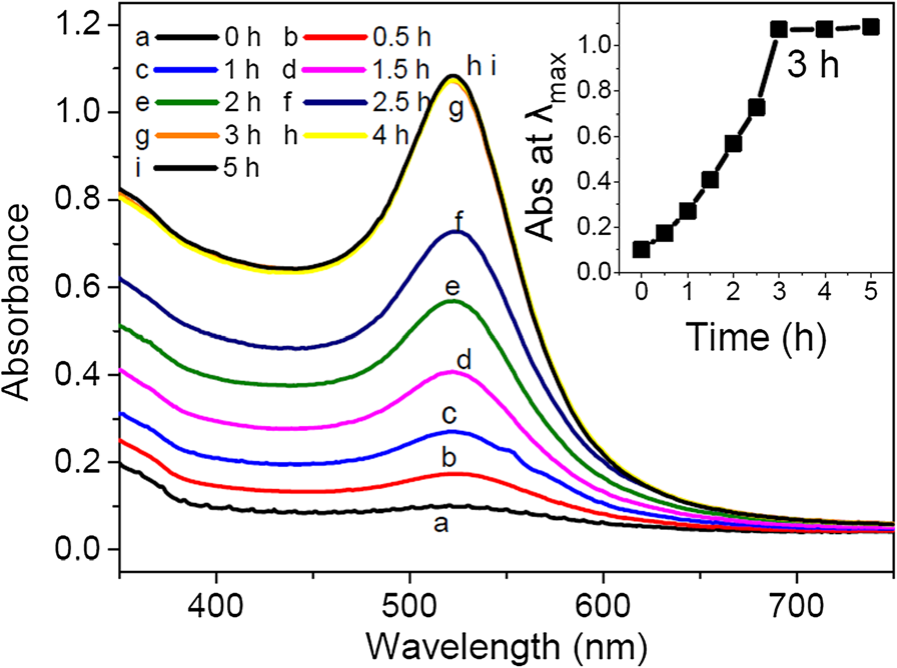
**UV-vis spectra of gold nanoparticles synthesized by KGM after incubation at 50°C for different times.** The final concentrations of HAuCl_4_ and KGM are 0.89 mM and 0.22 wt%, respectively. The inset presents the reaction kinetics for the formation of gold nanoparticles.

The formation of gold nanoparticles by reduction of HAuCl_4_ with KGM was investigated by UV-vis spectra at different reaction times. As confirmed by kinetic measurement of the spectra (Figure 2), the intensity of the absorption peak increased gradually with time and reached a maximum after 3 h which means that the reaction has reached saturation. The reaction seems to reach saturation abruptly as shown in the inset of Figure 2. The possible reason is that the growth process of KGM-capped gold nanoparticles was complicated since there are various interactions occurring simultaneously. Specifically, KGM was employed both as reducing and stabilizing agent for the synthesis of gold nanoparticles.

As shown in Figure 2, all spectra exhibit an absorption peak around 522 nm with no significant peak shift, which is attributed to the surface plasmon resonance (SPR) band of the AuNPs, indicating the formation of gold nanoparticles. During the formation of AuNPs, the color of the reaction mixture changed from colorless to light pink within approximately 0.5 h and finally to wine red after 3 h. The phenomenon of color changes revealed a nucleation-growth mechanism (which is different from the typical synthesis of gold nanoparticles in citrate reduction) according to Ji et al.'s work [30], which would be discussed later.

**Figure 3:**
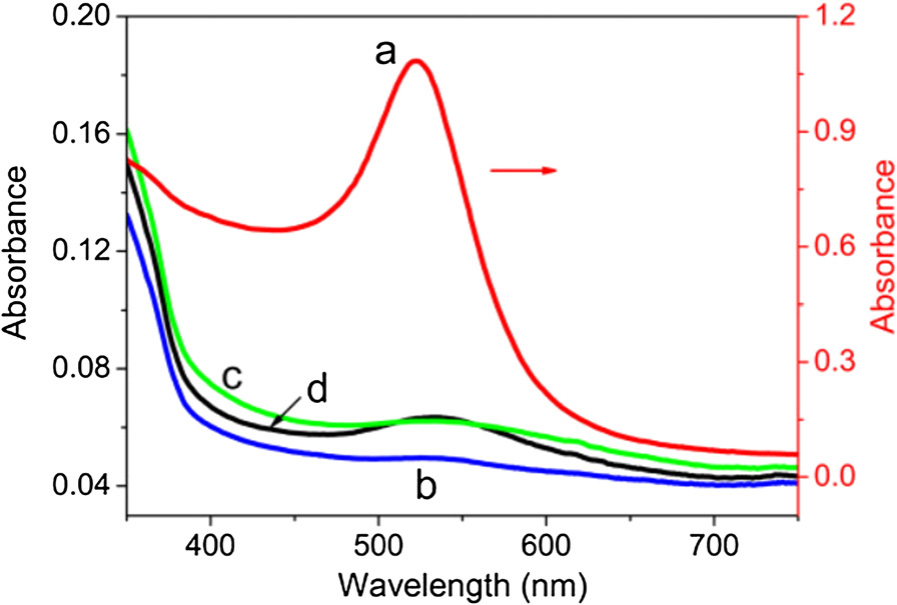
**UV-vis absorption spectra for AuNPs.** (a) Under optimized conditions: 0.89 mM HAuCl_4_ and 0.22 wt% KGM in NaOH solution at 50°C for 3 h. (b) In the absence of NaOH, with other conditions the same as in (a). (c) With 0.89 mM HAuCl_4_ and 0.22 wt% KGM in NaOH solution at 25°C for 12 h. (d) AuNPs synthesized with 0.89 mM HAuCl_4_ and 0.1 wt% KGM in NaOH solution at 50°C for 3 h.

There were several influencing factors in the biosynthesis process. It was noted that alkaline addition (sodium hydroxide in our work) was necessary for the formation of gold nanoparticles. As shown in Figure 3 (curve a), AuNPs were obtained in alkaline solution. If no NaOH or insufficient NaOH was added to the reaction system, KGM failed to reduce gold precursor salts as a result of its weak reduction ability under acidic, neutral, or weakly basic conditions. A control experiment without adding sodium hydroxide was performed in the same reaction conditions as in the synthesis of AuNPs (Figure 3, curve b). The reaction temperature was also another important factor. It was found that the reaction was extremely slow at 25°C, at which no nanoparticles were detected after 12 h of reduction (Figure 3, curve c). When conducted at a temperature higher than 80°C, the reaction was completed within less than 30 min. However, some visible aggregates were observed due to the gelation of KGM in alkaline solution when temperatures were higher than 55°C [31]. Therefore, we conducted the reactions at 50°C at which it showed a reasonable reaction rate. In addition, the concentrations of KGM and gold precursor were also critical. At a fixed gold precursor concentration (0.89 mM), a high KGM concentration (0.2 to 0.5 wt%) was required for the effective formation of AuNPs. Decreasing the KGM concentration to 0.1 wt%, while keeping the gold precursor concentration constant (0.89 mM), would produce very little nanoparticles with a weak SPR peak (Figure 3, curve d). The solution of dispersed gold nanoparticles in KGM was highly stable and showed no signs of aggregation after 3 months of storage. Besides, we also examined the stability of the as-synthesized gold nanoparticles under different pH values. No obvious change in UV-vis spectra was observed for AuNPs in solutions of a broad pH range (3 to 13), adjusted by adding hydrochloric acid or sodium hydroxide. The high stability of the prepared nanoparticles would greatly facilitate their use in some biological applications.

### Analysis of morphologies and crystalline structure of AuNPs

**Figure 4:**
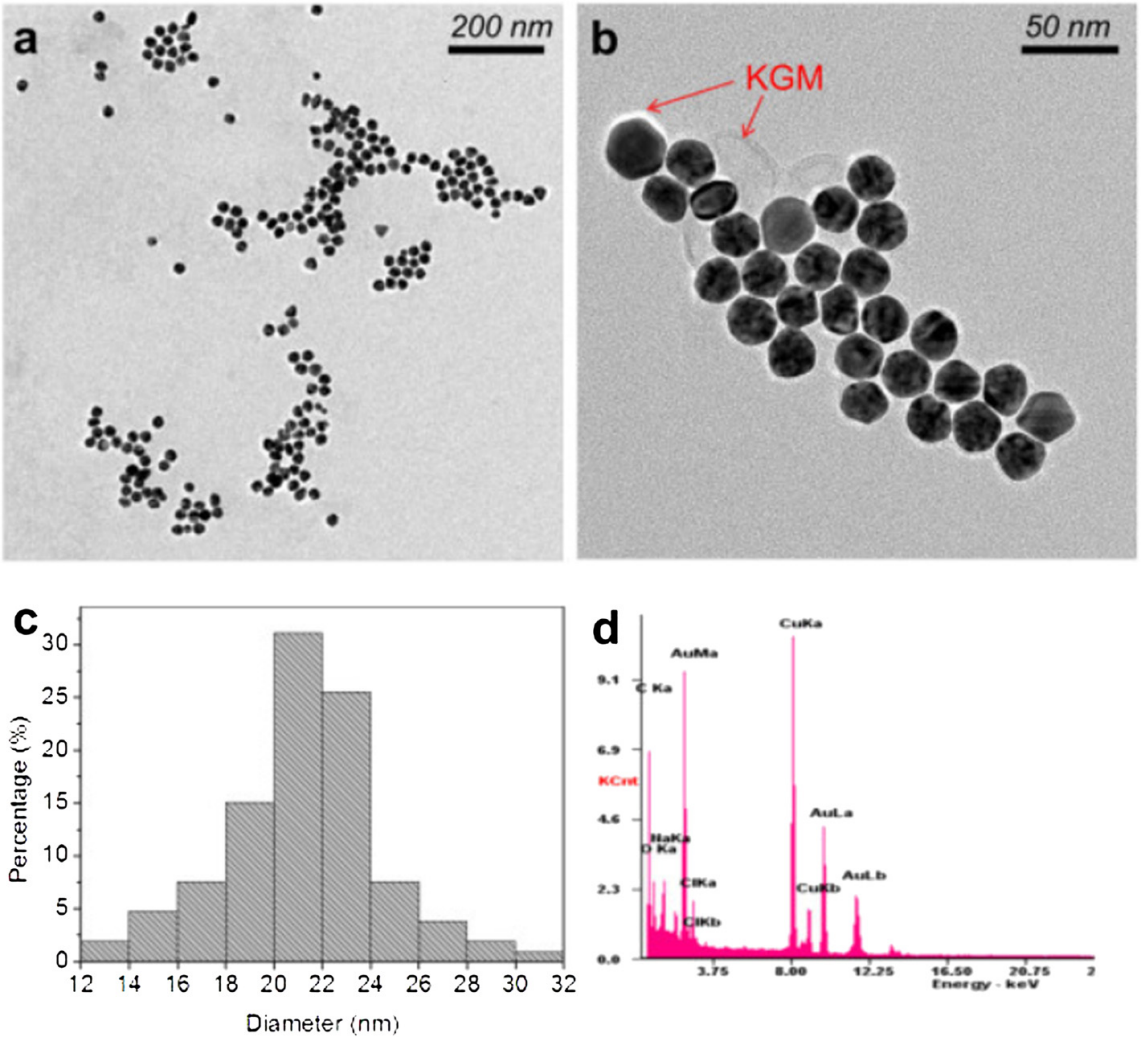
**TEM images and EDAX spectra.** TEM images of the **(a**, **b)** morphology of the AuNPs and **(c)** the corresponding particle size distribution of AuNPs. **(d)** EDAX spectra of AuNPs.

The size and shape of the synthesized AuNPs were confirmed by TEM analysis. Typical TEM images of the nanoparticles formed were presented in Figure 4a,b, which show that the gold nanoparticles exhibit uniform spherical shape. Figure 4c presents a histogram of particle size distribution by manual analysis of 203 particles viewed from TEM images. The particle sizes distribute in the range of 12 to 31 nm, with the mean particle diameter = 21.1 nm and *σ* = 3.2 nm. More than 80% of the particles are in the range of 21.1 ± 5 nm, indicating a relatively narrow distribution of the AuNPs formed in this work. As shown in Figure 4b, it could be clearly seen that the AuNPs were coated with a layer of KGM with a thickness of 2 to 3 nm, suggesting the stabilizing effect of KGM for AuNPs. The EDX result demonstrated strong peaks of Au at 2.195 keV and also confirmed the existence of C and O indicating the adsorption of KGM on the surface of the gold nanoparticles. The Cu signals were due to the use of a copper grid, and the appearance of Cl was caused by the existence of AuCl^4-^ ions.

**Figure 5:**
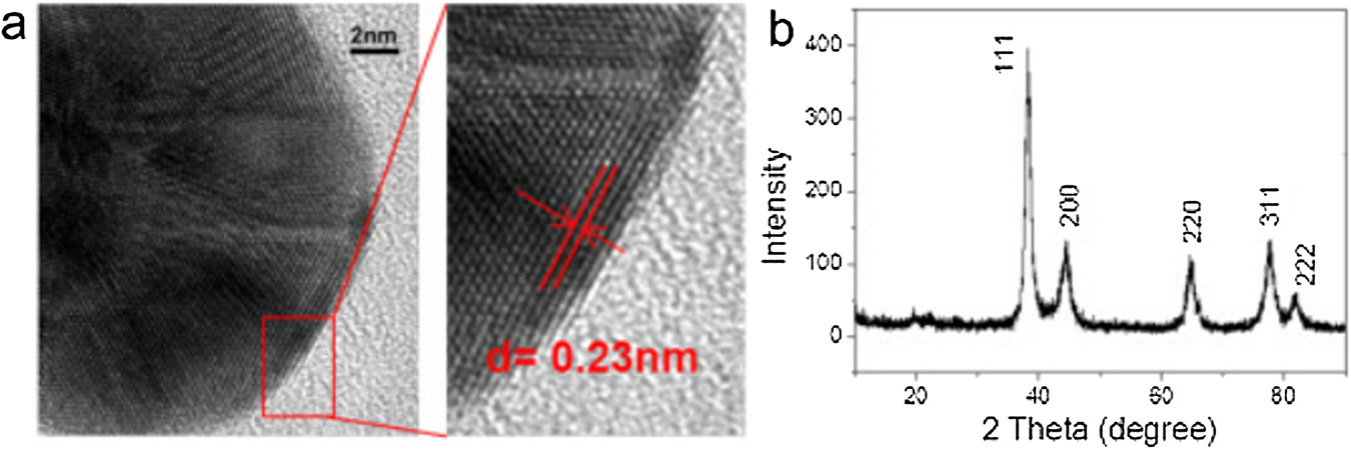
**Gold nanoparticles formed in the system. (a)** High-resolution TEM images and **(b)** XRD pattern.

The crystalline structure of the prepared nanoparticles can be illustrated using high-resolution TEM (HRTEM) and XRD. The HRTEM images shown in Figure 5a exhibit clear lattice fringes with interplanar spacing of 0.23 nm corresponding to the (111) planes of the face-centered cubic (fcc) AuNPs, confirming the formation of polycrystalline gold nanoparticles. Furthermore, the XRD pattern of freeze-dried gold nanoparticles (Figure 5b) showed that the diffraction peaks were located at 2*θ* = 38.55° (111), 44.90° (200), 65.07° (220), 77.86° (311), and 81.86° (222) attributed to gold nanoparticles, thus further proving the fcc structure of AuNPs in the system.

### Mechanism analysis by FTIR study and DLS

**Figure 6:**
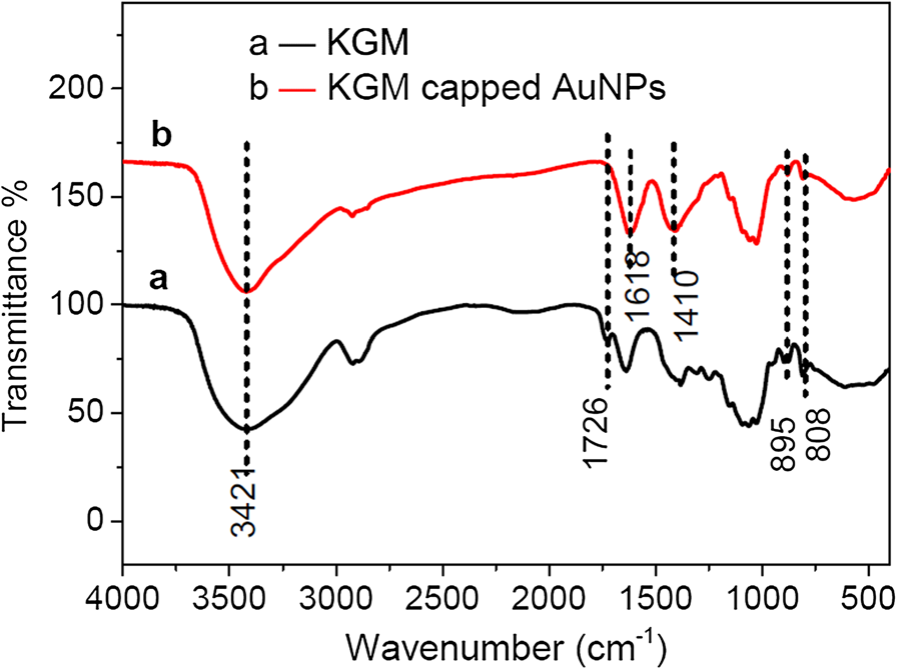
FTIR spectra of (a) pure KGM and (b) freeze-dried gold nanoparticles synthesized in KGM solutions.

FTIR spectra of pure KGM and freeze-dried AuNPs prepared in the KGM solution were recorded to investigate the interaction between gold nanoparticles and KGM. KGM consists of β-1,4-linked d-mannose and d-glucose in the ratio 1.6:1, with about 1 in 19 units being acetylated. Accordingly, as shown in Figure 6a, KGM exhibited a characteristic absorption peak of the β-1,4-linked glycosidic bond at 895 cm^-1^ and a characteristic peak of the enlargement of pyranoid rings at 808 cm^-1^[32]. In alkaline solution, the deacetylation of KGM occurred, which resulted in the disappearance of the peak at 1,726 cm^-1^ corresponding to the group of C = O, consistent with the previous wok of Maekaji [33]. Here, KGM plays the role of both reducing agent and stabilizer in the process. The FTIR spectra provide evidence for the role of reducing agent. The relatively strong absorption bands observed in the FTIR spectrum of the AuNPs (Figure 6, curve b) at 1,618 and 1,410 cm^-1^ coincide with the carboxylate (Au-COO^-^) groups. Here, the hydroxyl groups of KGM act as the reducing species for the reduction of Au^3+^ ions into Au^0^, and they were oxidized into carboxylic acid.

Besides, the stabilizing effect was also confirmed by FTIR spectra. As shown in Figure 5, the absorption peak in the area of 3,421 cm^-1^ arose due to O-H stretching vibrations of the hydrogen-bonded hydroxyl (OH) group. A remarkable difference between the curves for pure KGM and KGM-protected AuNPs was the narrowing at 3,421 cm^-1^ (Figure 6, curve b). The narrowing of this peak was due to the damage of hydrogen bonding of the hydration between the KGM molecular chain and the water molecule in alkaline solutions [31,34]. Thus, the formation of free -OH group facilitates the coordination interaction with gold ions by the breaking of hydrogen bonding. Taken together, the FTIR results demonstrate that initially gold ions bind to the surface of the KGM molecules and are subsequently reduced by hydroxyl groups, leading to the generation of nucleation sites for further reduction and ultimately to the formation of gold nanoparticles. The *in situ* reduction process prevents the aggregation of AuNPs.

### Formation mechanism of gold nanoparticles in aqueous KGM solution

**Figure 7:**
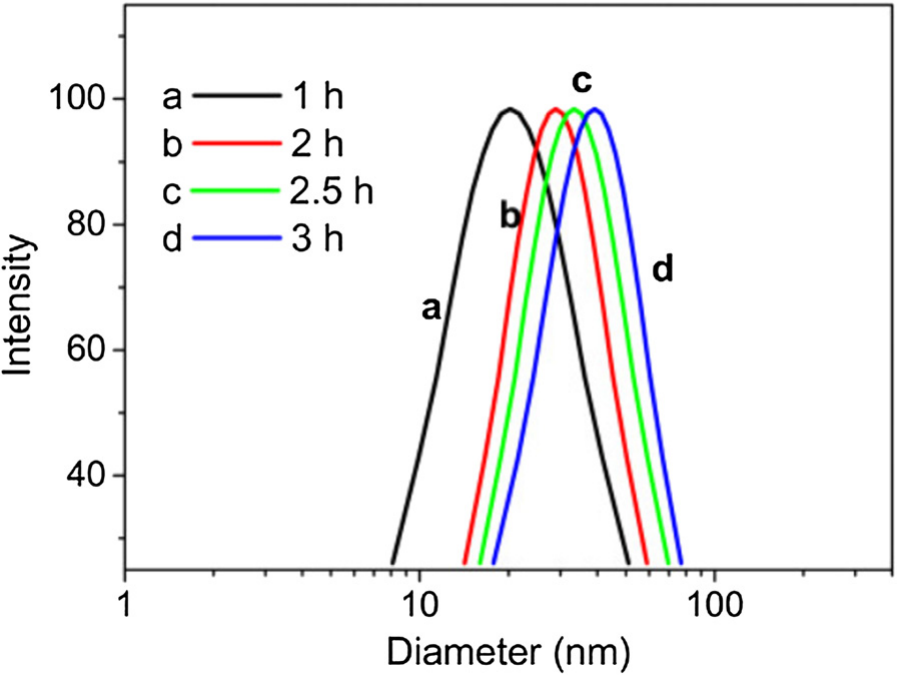
**Size distribution of gold nanoparticles at different reaction times.** Reaction condition: with final concentrations of HAuCl_4_ and KGM to be 0.89 mM and 0.22 wt%, incubated at 50°C.

Typical synthesis of gold nanoparticles by citrate reduction in Frens' method, which was mostly used, is formed though a nucleation-aggregation-smoothing pathway [30]. As mentioned before, the reaction here was completed through a nucleation-growth route. In order to gain further insight into the mechanism of nanoparticle formation, dynamic light scattering was employed to investigate the size change in the reaction process. As shown in the DLS results (Figure 7), with the reaction time increasing, the hydrodynamic diameter increased from 20.3 to 39.2 nm, which means that the particles grew gradually in the reaction. The synthetic approach described in this study avoided the nanowire aggregates as the intermediates in the middle step of typical citrate reduction in Frens' method [4,30]. Thus, the as-synthesized nanoparticles exhibited a uniform, relatively narrow size distribution.

In our work, KGM was employed both as reducing agent and stabilizer for the synthesis of gold nanoparticles (Figure 1). Here, abundant hydroxyl groups of KGM act as the reducing groups for the reduction of Au^3+^ ions to Au^0^. It is worth noting that the deacetylation and cross-linking of KGM following alkali addition play an important role. The alkali damaged the hydrogen bonding of the hydration between the molecular chain and water molecules [35], resulting in the formation of free -OH group along the KGM chains which play the role of reduction and stabilization. Due to deacetylation and cross-linking behavior, KGM macromolecules contain size-confined molecular level capsules, which can act as templates for nanoparticle growth. Raveendran et al. [23] reported a similar situation where starch served as a good template or dispersant for preparing well-dispersed nanoscale Ag particles in aqueous media without agglomeration. Thus, our results showed that it may be possible to achieve better size distribution control of the nanoparticles and good dispersity by selecting the appropriate reductant and stabilizer from various biological materials. In conclusion, the AuNPs formed in the KGM solution could be stabilized by a combination of gold-hydroxyl interaction and the steric stabilization owing to the molecular-scale entanglement of the polysaccharide.

### Catalytic properties

Transition metal nanoparticles are attractive to use as catalysts due to their high surface-to-volume ratio compared to bulk catalytic materials. To date, the use of metal nanoparticles synthesized with polysaccharide is very limited. Here, our TEM images above showed that the gold nanoparticles are nearly spherical in shape and are composed of numerous (100) and (111) planes with corners and edges at the interfaces of these facets. Hence, the as-prepared gold nanoparticles are expected to be catalytically active. To investigate their catalytic activity, the reduction of 4-NP to 4-AP by NaBH_4_ was selected as a model system. It is well known that the absorption spectrum of a mixture of 4-NP and NaBH_4_ shows an absorption peak at 400 nm corresponding to the formation of an intermediate 4-nitrophenolate ion. Thus, the reaction process can be monitored by monitoring the changes in the absorption spectra of the 4-nitrophenolate ion at 400 nm. In a control experiment without AuNP addition, the absorbance at 400 nm did not change with time, indicating that no reduction of 4-NP occurred in the absence of AuNPs. Immediately after addition of nanoparticles, there was a remarkable decrease in the intensity of the absorption peak at 400 nm, and at the same time, a new peak at 298 nm appeared indicating the formation of reduction product, 4-AP.

**Figure 8:**
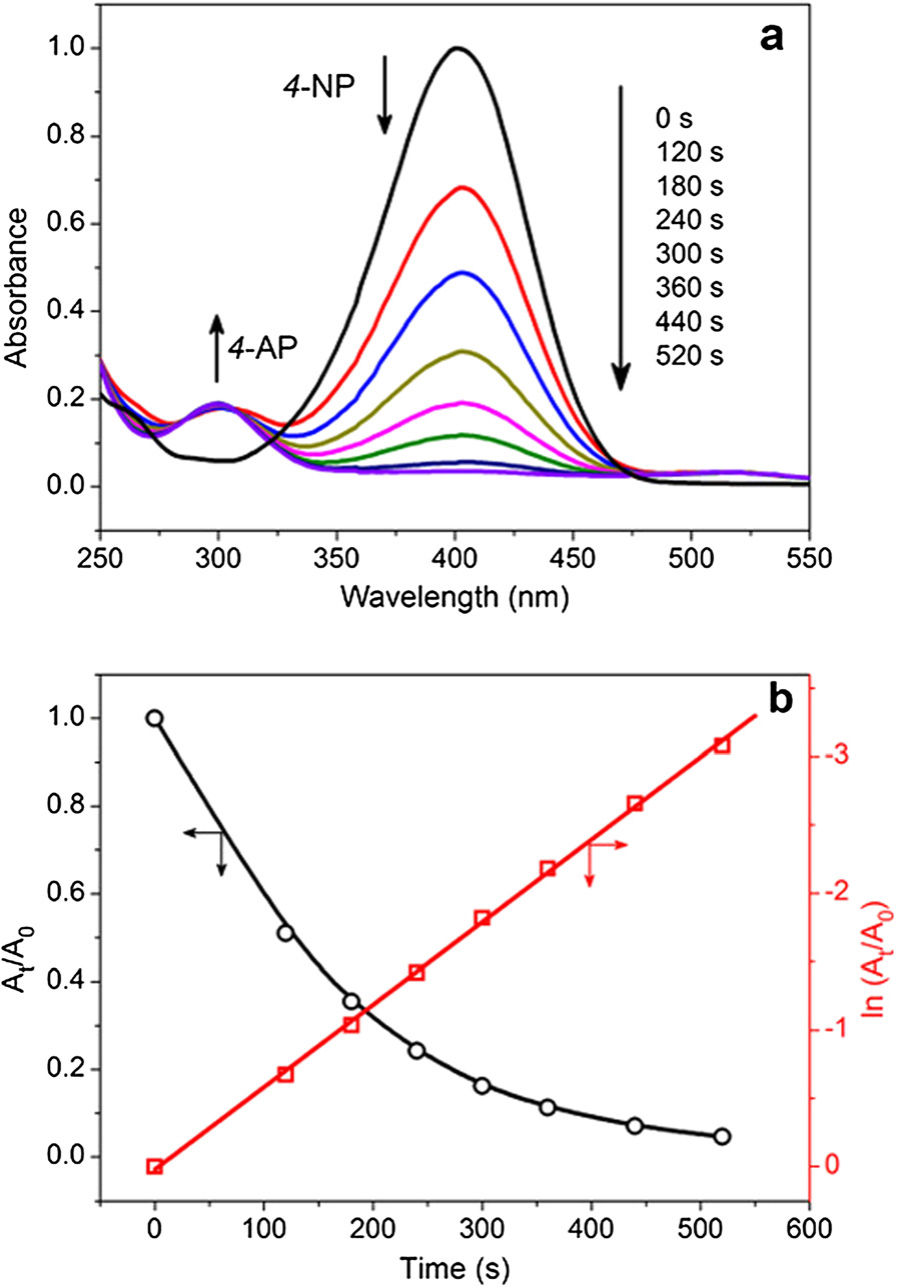
**Absorption spectra and plots of ln *A***_***t***_**/*A***_**0 **_**and *A***_***t***_**/*A***_**0 **_**versus time. (a)** Time-dependent UV-vis absorption spectra for catalytic reduction of 4-NP by NaBH_4_ in the presence of AuNPs. **(b)** Plots of ln (*A*_t_/*A*_0_) and *A*_t_/*A*_0_ versus reaction time for the reduction of 4-NP; *A*_0_ and *A*_*t*_ were the absorption peak at 400 nm initially and at time *t*. Condition used throughout: [4-NP] = 0.5 × 10^-4^ M, [NaBH_4_] = 1.0 × 10^-2^ M, and *T* = 25°C.

Figure 8a shows time-dependent absorption spectra of the reduction with the obtained gold nanoparticles. The results showed that the KGM-capped gold nanoparticles can successfully catalyze the reduction reaction. It could be observed that the reaction was almost completed within 600 s in the presence of NaBH_4_ (Figure 8a). Since the concentration of sodium borohydride far exceeds the concentration of 4-NP, the reduction rate can be assumed to be independent of the borohydride concentration. In this context, a pseudo-first-order rate could be used to evaluate the kinetic reaction rate of the current catalytic reaction. Figure 8b shows the plot of ln *A*_*t*_/*A*_0_ and *A*_*t*_/*A*_0_ versus time. ln *A*_*t*_/*A*_0_ decreased linearly with reaction time, indicating that the reduction reaction follows first-order kinetics. The first-order rate constant was calculated to be 6.03 × 10^-3^ s^-1^, and this value shows that the AuNPs prepared here with KGM possess better catalytic activity compared to other polysaccharides and some extracts (Table 1). In addition, the rate constant is comparable to that of other biologically synthesized gold nanoparticle catalysts for the reduction of 4-NP in the presence of NaBH_4_. The catalysis of the gold nanoparticles is possibly due to the efficient electron transfer from the BH^4-^ ion to nitro compounds mediated by the nanoparticles. This could be attributed to the higher driving force of particle-mediated electron transfer caused by their large Fermi level shift in the presence of highly electron-injecting species such as borohydride ions.

**Table 1:** Recent studies on the reduction of 4-NP with biologically synthesized AuNPs

**Composition**	***T *** **(K)**	**Size (nm)**	**Rate constant (s**^**-1**^**)**
α-Cyclodextrin-coated AuNPs [36]	298	11 to 26	2.98 to 4.65 × 10^-3^
Au-calcium alginate composite [37]	291 to 306	5 ± 2	0.23 to 0.33 × 10^-3^
AuNPs synthesized with fruit extract (*Prunus domestica*) [38]	298	4 to 38	1.9 to 5.1× 10^-3^
AuNPs synthesized with protein extract (*Rhizopus oryzae*) [39]	303	5 to 65	2.81 to 4.13× 10^-3^
KGM-synthesized AuNPs (this work)	298	12 to 31	6.03 × 10^-3^

## Conclusions

In this study, we describe a facile and economically viable route for the synthesis of well-dispersed spherical gold nanoparticles using konjac glucomannan. The synthesized nanoparticles exhibit uniform spherical shape, a narrow size distribution with a mean diameter of 21.1 ± 3.2 nm, and excellent stability after 3 months of storage. The morphology and crystalline structure were characterized by TEM and XRD. Furthermore, the formation mechanism of AuNPs and the role of KGM both as reducing agent and stabilizer were analyzed by the results of UV-vis, TEM, DLS, and FTIR. Finally, the as-prepared gold nanoparticles were found to serve as effective catalysts for the reduction of 4-nitrophenol in the presence of NaBH_4_. Our work promotes the use of natural polysaccharide for the biosynthesis of nanomaterials, and more efforts should be made to extend their applications in biologically relevant systems.

## Competing interests

The authors declare that they have no competing interests.

## Authors' contributions

ZG and RXS designed the research. ZG performed the research. ZG, RXS, RLH, WQ, and ZMH analyzed the data and wrote the paper. All authors read and approved the final manuscript.
